# Towards big data behavioral analysis: rethinking GPS trajectory mining approaches from geographic, semantic, and quantitative perspectives

**DOI:** 10.1007/s44223-022-00011-y

**Published:** 2022-07-26

**Authors:** Weixin Huang, Luying Wang

**Affiliations:** School of Architecture, Tsinghua University, 30, Shuangqing Road, Haidian District, Beijing, 100084 China

**Keywords:** Behavioral analysis, Clustering, GPS trajectory mining, Sequence alignment, The built environment, The Palace Museum

## Abstract

The question regarding the actual usage of built environments is of immense importance in behavioral research. Yet traditional methods of collecting and analyzing data on movements and activities often lack needed accuracy and granularity. Thus, this article reviewed and summarized the applicability of emergent GPS trajectory mining approaches in the field of architecture from geographic, semantic, and quantitative perspectives, respectively. Accordingly, three experiments based on a case study using real GPS trajectory data from visitors to the Palace Museum in China were conducted to examine the usefulness and weakness of the aforementioned approaches. The findings revealed that although all three dimensions of the trajectory mining approaches had the potential to provide useful information for architectural and urban design, the higher the dimensionality in utilizing the data, the more effective the approach was in discovering generalizable knowledge of human behavioral pattern. Furthermore, the results suggested that to gain insights into the typological characteristics of human behaviors related to the built environments, the contribution of trajectory data alone was limited, hence, conventional field surveys and questionnaires which contain information on individual characteristics and spatial features should be used in conjunction. Future research and practical implications were outlined.

## Introduction

The relationships between behavioral science and the built environment are of growing importance during recent decades, since awareness of rational allocation of spatial and environmental resources has risen. Mobility behavioral analysis specifically focuses on space utilization and users’ spatial-temporal behavioral patterns, and contemporary mobility behavioral typology studies have an increasing field of interest on this issue. In the field of architecture, most of the research emphasizes flow organization and functional structure in architectural spaces (Yang & Huang, 2019; Sheng et al., 2021), while in the behavior domain, individual characteristics and demands on the usage of spaces are worth more exploration (Kupper & Seyfried, 2020; Xu et al., 2020). For the recording of human mobility behavior, there are different data sources and methods, including activity diaries (Wilson, 2008), qualified observations (Peng & Maing, 2021), questionnaire surveys (Lee et al., 2011), spatial syntax (Chang & Penn, 1998; Yang et al., 2015; Sheng et al., 2021) and a variety of visualization attempts (Zhang et al., 2021). These methodologies are standardized nationally and internationally by organizations such as the International Social Science Council (ISSC) and the Chinese Academy of Social Sciences (CASS) as behavioral measurement and interpretation tools and are also widely practiced in architecture and urban space investigations. Nevertheless, conventional qualitative methods are too limited and restricted to answer complicated questions about actual space usage in the built environment. The traditional questionnaire survey on space usage behavior, for example, such as the evaluation of flow organization and spatial layout, does not provide researchers with information concerning the real-time trajectories of the users or variations in behavioral decision-making when engaging with the environment. Self-report measures based on participants’ feelings and memories are also prone to information omissions and impression mistakes. Qualified observations from a spectator’s perspective could to some extent cover these deficiencies, but it is scarcely possible to monitor a user’s spatial-temporal activities permanently with limited numbers of observers. Spatial-temporal behavioral analyses play a more and more important role in humanizing flow design, facilitating sustainable public resource allocation, and boosting post-occupancy evaluation. Data obtained following the traditional methodology, however, remains too sparse in resolution, and the majority of spatial behavioral studies still tend to follow qualitative approaches or focus on very narrow case studies. This has prompted an extensive debate on the scope and methodological innovation in research examining human behavior related to the built environment (Kwan et al., 2003; Hadjri & Crozier, 2009; Zhang et al., 2021).

Spatial-temporal behavioral studies in architectural and urban design contexts, despite the comprehensiveness of qualitative approaches, still need quantitative datasets to recognize the contradictions between the pre-designed spatial flows and the actual movement behavior of space users. Further, data collection techniques without interfering with individual activities are necessary to record individual movement trajectories over time objectively and accurately. Recent developments in space positioning technology, including Wi-Fi indoor positioning system (IPS), ultra-wideband (UWB) IPS, Bluetooth technology, mobile (cellular) phone positioning, global positioning system (GPS), along with multi-source big data including geo-tagged photos and surveillance videos are advancing surveying methods and digital datasets in spatial-temporal behavioral studies. The geographical information system (GIS) and relevant visualization methods also contribute to time and space behavioral studies (Ahas et al., 2008; Wu & Carson, 2008; Kwan et al., 2015; Xu et al., 2020). One of the emerging subjects in movement behavioral studies is connected with GPS trajectory mining and spatial-temporal sequence analysis (Cho & Kang, 2019; Li et al., 2021; Tang et al., 2018; Yuan et al., 2017; Brum-Bastos et al., 2018). GPS trajectory mining has great potential for applications in space-time behavioral studies in buildings and urban spaces, though there are various restrictions and inaccuracies when applied in indoor spaces. Nevertheless, GPS trajectory data tends increasingly to complement conventional data sources and behavioral observations, as it is becoming crucial how humans utilize space and public resources.

GPS trajectory data is stored automatically in the memory files of handheld GPS locators, containing a huge amount of geographical information, which requires specialized analytic tools (e.g. GIS, Python programming, etc.) and data mining algorithms to obtain useful patterns and knowledge. The objective of this paper was to introduce and evaluate the applicability of the GPS trajectory mining approaches, which have been explored mainly in transportation and tourism studies but seldom studied in behavioral science. Three main research questions are as follows:

1. What are the potentials of trajectory mining approaches for uncovering useful behavioral patterns in architectural and urban design?
2. How do different trajectory mining approaches perform? Which approach is more suitable for exploring behavioral patterns in complex spatial-temporal trajectories?
3. With GPS trajectory data and mining techniques, do conventional data and methods such as questionnaires still matter?

To gain these insights and determine the potential worth of the various trajectory mining approaches, we illustrated our discussion with three analytical examples from the Palace Museum study projects with visitors’ GPS trajectory data during the tour, and the advantages and limitations of each trajectory mining approaches were explored and discussed. The first example tested a hierarchical raw-data-based clustering method to identify the spatial similarity in the visitors’ GPS trajectories from a geographic perspective. The second example examined a supervised sequence alignment approach to recognize similar trajectory sequences from a semantic perspective. The third example evaluated the metrics-based clustering approach, which used three spatial-temporal measures to represent movement features to cluster the trajectories, and the characteristics of clustering results were explored.

## Trajectory pattern mining and behavioral science of the built environment

The study of human behavior in relation to the built environments and post-occupancy evaluation are quite intertwined, and these two research fields both encounter the same challenge: a lack of suitable technical tools for measuring and collecting data regarding the actual usage of the built environments. As Cooper et al. (1991) mentioned in a paper,” the question of measurement is difficult”, and “a data bank for the dissemination of information on accessibility and other aspects of environmental assessment” should be developed. To address this question, trajectory datasets, including those without high resolution as GPS, could play a significant role, because behavioral patterns could be identified from these trajectories, and provide reliable empirical evidence for post-occupancy evaluation and behavioral analysis related to the built environment.

More specifically, the GPS trajectory raw data contains basic information including time points, latitude, longitude, and altitude coordinates. Aside from trajectory visualization and basic spatial-temporal distribution, it is necessary to further mine the trajectory to obtain the available knowledge and implicit behavioral patterns. For example, identifying the frequent trajectory segments or location sequences in trajectories could provide insights on questions like which paths and spaces in a place have higher usage rates, and how to allocate public resources reasonably in line with the amount of people’s usage demand.

Take another example of stay behavior, identifying the stopover points or short stay areas from the trajectories could help identify the consistent stay behavior of people, so as to know which places or areas are more likely attractive for people to stay. Furthermore, the spatial and landscape features including spatial openness, spatial shape, interfacial textures, and the presence of greenery in these stay areas could be examined. In addition, analyzing the diverse patterns of people with diverse characteristics in stay behavior provides us the opportunity to further understand human decision-making mechanisms, and this knowledge obtained from the trajectory tends to be useful for the design of the architectural and urban spaces.

The exploration of trajectory data and its mining approaches for behavioral studies in built environments has been increasingly significant and pressing since the outbreak of the COVID-19 pandemic (Lu et al., 2021). In a special issue of *Architectural Journal*, there was a group discussion about the impact of COVID-19 on the contemporary city, new human habitat, and architectural design led by three Chinese academicians and 22 experts, scholars, and architects (Wang et al., 2020). In this paper, a lack of corresponding empirical data and documentation on spaces formed by temporary gatherings, including bazaars and food markets was pointed out. Previous researchers have hardly examined the flow distribution, human behavioral characteristics, and the relationships between main flow lines during the usage of such spaces, resulting in difficulties in making rational judgments and accurate decisions when encountering unexpected events like the COVID-19 pandemic. When tracking the movement of patients to identify potentially infected persons, if provided with a more complete database on human spatial behavior, key associated areas and vulnerable populations could also be predicted and the virus transmission chain could be stopped in advance.

The demands for human trajectory data in green building and the building industry are also emerging, particularly in the context of sustainability and smart buildings (Moreno et al., 2016; Chen et al., 2019). Making predictions of human behavior or occupation and including these considerations in the design phase before construction or in the building control system can help with building energy saving and hence carbon reduction. With digital positioning data, the feasibility of such predictions has been confirmed, although relevant research results are limited in number (Jin et al., 2021). An occupancy prediction model was proposed by Huang et al. (2019) using a Bayesian method and proved to be effective based on the dwell time distribution extracted from the passenger flow data in a Chinese hub airport terminal.

Individuals’ trajectories not only reflect the places they visited, the routes they took, or the locations they stayed for a long period but also contain certain implicit information. For example, it is possible to identify people who may be companions based on the spatial-temporal similarity of trajectories, to discover the spatial functional associations based on frequent trajectory sequences, or to demonstrate the consistency of spatial-temporal behavioral patterns based on certain clustering features, even though their trajectory routes may be rather diverse. Further, we could discover how people make behavioral decisions regarding the utilization of spatial and temporal resources, and what factors may affect certain unconscious behavioral decisions.

All examples mentioned above demonstrate the importance of trajectory data in achieving an insightful understanding of human movement behavior in architectural or urban spaces. To the best of our knowledge, few studies, however, have addressed the aforementioned issues from the perspective of architectural disciplines involving the use of high-resolution trajectory data. While relating to all issues is beyond the scope of a single article, this study, therefore, tends to bring up a framework that structures an original synthesis of trajectory pattern mining and behavioral research within the built environments, whose feasibility will be proved with a case study focusing on the evaluation of the applicability of various trajectory mining approaches.

## Three dimensions of trajectory mining approaches

Early applications and analysis of GPS trajectory data originated from the field of transportation (Chakka et al., 2003) due to the application of GPS navigation systems in vehicles since the year 2001, producing massive vehicle and drivers’ movement data. Two important tasks for trajectory data mining involve identifying similar paths and potential clusters in trajectories (Andrienko et al., 2008), hence, relevant approaches and algorithms are constantly being explored and innovated by numerous researchers and practitioners (Zhou et al., 2005, 2019; Lee et al., 2007; Dodge et al., 2012; Yuan et al., 2017; Cheng et al., 2021). Considering existing trajectory data analysis applications, the approaches of trajectory mining could be categorized into the following three types: raw-data-based, sequence-based, and metrics-based, interpreting the trajectory data in three diverse dimensions.

### Raw-data-based approach

Raw GPS positioning data are usually of high resolution and massive data volume, and subject to occasional point jumps, which make it necessary to preprocess the trajectory data before analysis, otherwise, it would result in excessive memory consumption and unacceptable running time if we calculate the distance similarity for two trajectories directly. For this reason, trajectory data preprocessing techniques including compression, filtering, segmentation, and transformation were one of the most important research directions (Zheng & Zhou, 2011), aiming at efficient distance similarity calculation and fast clustering while retaining adequate geographical information (Lee et al., 2007; Tang et al., 2018). With the involvement and contribution of researchers in the field of computer science, relevant algorithms and techniques have been developed and exploited deeply (Lee & Krumm, 2011), including the Douglas-Peucker (DP) algorithm for trajectory compression (Douglas & Peucker, 1973; Hershberger & Snoeyink, 1992) and the Kalman filter for trajectory smoothing (Gelb, 1974). Regarding the situation that the trajectories are similar in spatial but inconsistent in time, certain algorithms modified from the DP algorithm such as a top-down time-ratio (TD-TR) algorithm (Meratnia & de By, 2004) were proposed, which took into consideration both geographic and temporal features of the trajectories.

**Fig. 1:**
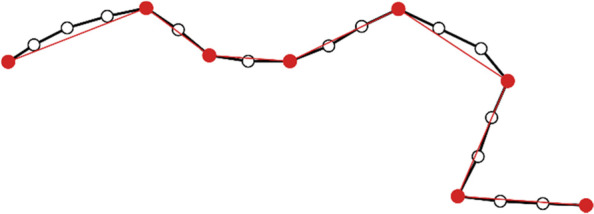
GPS trajectory segmentation and approximation according to important directional turning points

Raw-data-based trajectory mining approaches mainly attempt to resolve three major issues: frequent path search (Lee et al., 2007), trajectory route partition (Gaffney & Smyth, 1999), and stay area detection (Cho & Kang, 2019; Zhou et al., 2019). Regarding frequent path search, the basic analysis process is to segment the trajectories based on important directional turning points, where each segment represents an approximately linear path (see Fig. 1), and then calculate the similarity between segments based on certain features including distance and direction. After calculating the similarity matrix, various clustering algorithms including k-means, BIRCH, and DBSCAN could be used to search for similar trajectories. In the method framework of partition-and-group proposed by Lee et al. (2007), they argued that the density-based clustering method represented by DBSCAN is best suited for trajectory segment clustering because DBSCAN is particularly suitable for arbitrary shapes such as points and segments (Han et al., 2011).

**Fig. 2:**
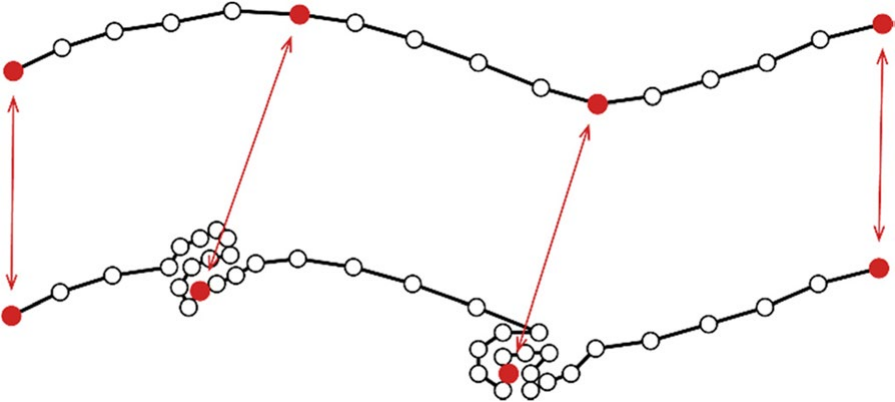
GPS trajectory resampling for distance similarity calculation

Unlike frequent path search, trajectory route partition focuses on the similarities between complete trajectories rather than trajectory segments, which means that not only the spatial features of trajectories but also the temporal features must be taken into account. The duration of similar routes might vary greatly due to diverse movement speeds, which leads to trajectories with spatial similarities containing a widely different number of points, making the similarity calculation result inaccurate. The key to the trajectory route partition task is therefore to find similar spatial patterns while eliminating the interference of temporal factors, where the idea of Dynamic Time Warping (DTW) (Berndt & Clifford, 1994; Müller, 2007) could be useful, an algorithm first applied to speech recognition, and currently used in many areas including data mining and time series clustering (Senin, 2008). DTW finds the optimal non-linear alignment between two time series, and one point in a time series could be mapped to multiple points in the other time series. Regarding GPS trajectory, the problem of spatial-temporal inconsistency could also be tackled in a relatively straightforward way by resampling the trajectories and converting them into series with the same number of points (see Fig. 2). This less rigorous approach is feasible because GPS devices usually have a fixed sampling rate, and the distance between each pair of sampling points is constrained without switching the transportation mode. Hence, the distance similarities are calculated only for the sampled points in order, which could greatly reduce the computational workload and improve the algorithmic efficiency.

**Fig. 3:**
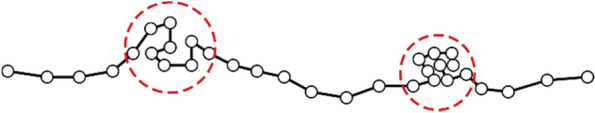
GPS trajectory points with high density as stay areas

Stay area detection is to identify various sets of points that appear in a small area consecutively for a period of time from movement trajectories, and it can be achieved by following two main concepts. One is to make full use of the spatial-temporal characteristics of the trajectory, calculate the average velocity of each trajectory segment, and mark a succession of points with an average velocity below a certain threshold as a stay area. The other approach is to treat all trajectories as a big dataset of points, and employ the density-based clustering algorithm (e.g. DBSCAN and OPTICS) to identify the points with high density as stay areas (see Fig. 3). This method, however, requires multiple iterations to adjust the parameters of DBSCAN regarding the neighborhood radius and the minimum number of points in each cluster, until an optimal stay area detection result is obtained.

### Sequence-based approach

The trajectory sequence is an alternative representation of the trajectory, which is composed of symbols converted from the original trajectory using semantic information. Compared with raw-data-based approaches, basic spatial shapes of trajectories are no longer considered, instead, a higher semantic dimension is extended. This makes sequence-based trajectory mining approaches suitable for addressing issues in tourism studies and behavioral science because the trajectory data in early tourism and activity research were mainly collected from travel or activity diaries (Wilson, 1998; Hallo et al., 2004), which contained sequence data of time, locations, and activities.

The question of how to analyze sequence data, however, was first raised in the field of molecular biology. To analyze the similarities between DNA or protein sequences, a sequence alignment method (SAM) was developed in the 1980s (Sankoff & Kruskal, 1983). SAM utilizes the edit distance such as the Levenshtein distance (Levenshtein, 1966) to measure the degree of similarity between two sequences. Edit distance or its variations (Dodge et al., 2012) are defined as the smallest number of elemental operations (e.g. insertions, deletions, and substitutions) required to convert one sequence into another. The more operations performed, the longer the distance between two sequences, hence, the smaller the similarity. The variations of SAM for GPS trajectory sequences could relate to artificially assigning different operation costs for different symbol pairs (Dodge et al., 2012), or representing GPS trajectories sequences using various coding methods, including meaningful functional locations, locations along with the time spent, and geographical polygonal area (Shoval et al., 2015).

Another common type of method for analyzing sequential data is association rule mining techniques such as PrefixSpan (Han et al., 2001), following the methodology of Apriori (Agrawal & Srikant, 1995), whose basic idea was to identify all of the strong association rules from the sequences given a minimum support threshold (the minimum occurrence frequency of the subsequences in the set of sequences) and a minimum confidence threshold (the minimum occurrence probability of the rule with a specific header subsequence).

A classical scenario using the Apriori method is to recognize patterns in sequences of events from an extensive database of customer transactions, for example, the rule {butter, bread} ⇒ {milk} means that if butter and bread are bought, customers also buy milk. With respect to trajectory sequences, certain fixed sequential patterns of associations between locations could be discovered using such approaches. In addition, the discovered rules could be used for predicting the next location of the following activity behaviors.

### Metrics-based approach

Trajectory metrics refer to various measures, including total distance, total time, movement direction, movement speed, movement efficiency, etc., which are derived or calculated from the trajectory data to describe and characterize the trajectory. The selection of measures is mainly determined according to research questions and the characteristics of sample data (Gaffney & Smyth, 1999; Dodge et al., 2012; Ferrante et al., 2018; Grinberger & Shoval, 2019). These measures constitute a multidimensional feature matrix of the trajectory and could be analyzed using various algorithms, including dimension reduction, classification, clustering, etc., which are commonly used in the field of artificial intelligence and data mining. These algorithms, though, will not be elaborated on in the article. Compared with raw-data-based and sequence-based approaches, the metrics-based trajectory mining approach could identify more abstract behavioral and activity patterns beyond geographic or semantic patterns, which reflects human behavioral characteristics rather than geographic or location-based features.

In this article, we based our analyses on the aforementioned three dimensions of trajectory mining approaches to fully explore the possibilities of GPS trajectory data. While this study uses the approaches that generally follow the existing concepts and schemes for trajectory pattern mining, no known studies have implemented these three schemes on the same dataset to compare their differences in knowledge discovery and to demonstrate their applicable research questions, which is quite important when introducing a set of new methodologies into an emerging research field.

## Case study: the Palace Museum

### Data overview

The case study was based on GPS trajectories of 503 visitors collected in the Palace Museum in Beijing, China during 4 days of independent field surveys in the summer of 2020. The temporal sampling rate of the raw GPS data was approximately one fix per 3 s. An elaborate pre-processing procedure was conducted, including parsing the original format of GPS data and filtering data with multidimensional constraints including space and time. After the pre-processing, 405 valid trajectories with information including time, longitude, latitude, moving distance, and moving speed were obtained. Further data processing and conversion were performed accordingly for three types of trajectory mining approaches respectively, and these steps will be elaborated on in the corresponding sections. In addition, it should be noted that all data processing, analysis, mining, and visualization procedures conducted in this paper were implemented using Python programming (Kuhlman, 2009).

### Raw-data-based approach: movement spatial similarity search from a geographic perspective

**Fig. 4:**
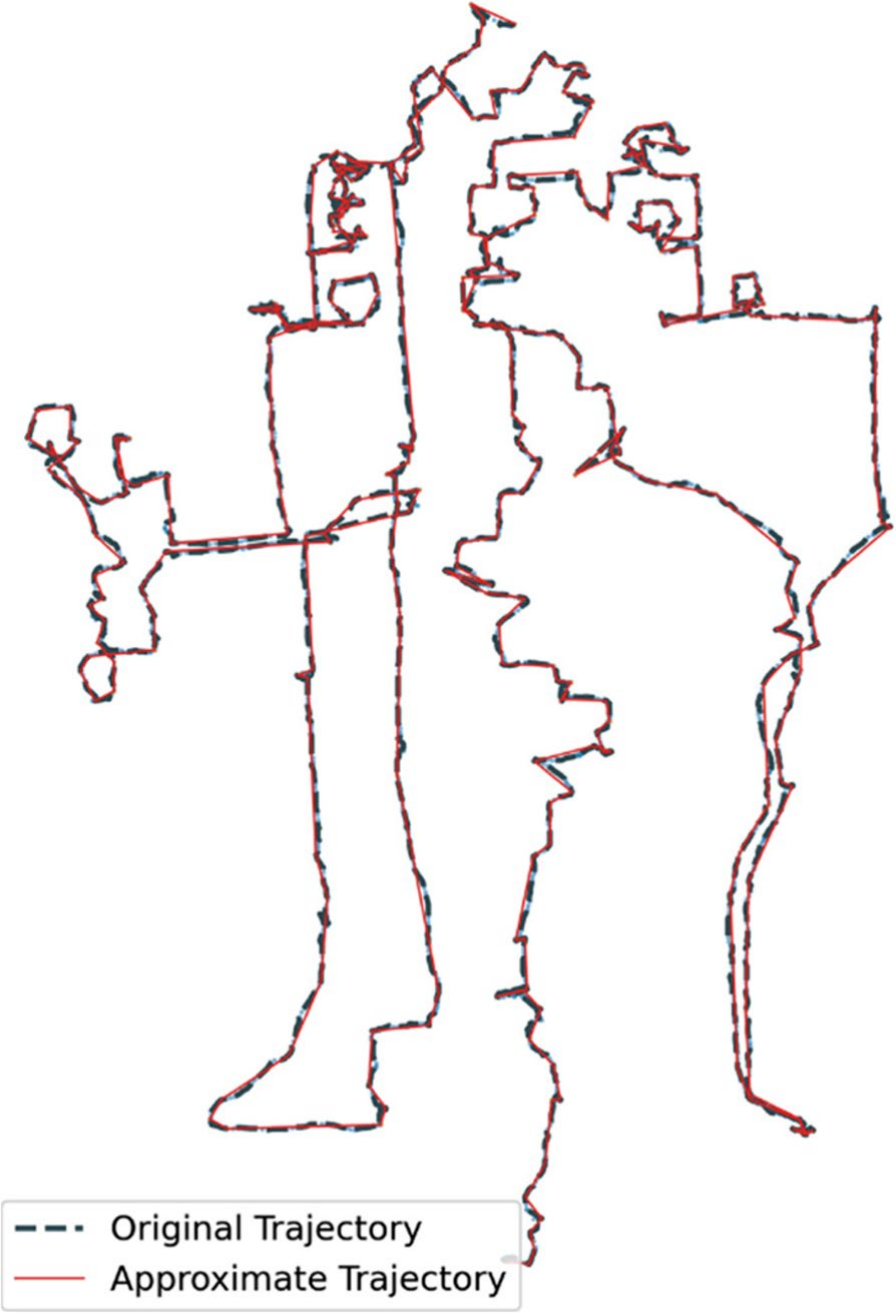
An example comparing the approximate trajectory with the original trajectory

This experiment was intended to test the raw-data-based trajectory mining approach, and a basic hierarchical clustering method using Euclidean distance was employed. To improve the efficiency of the algorithm operation, the Ramer-Douglas-Peucker algorithm (Lee et al., 2007) was applied following the pre-processing procedure for compressing and approximating the trajectories. An example of the approximate trajectory was drawn in Fig. 4, and the compressed trajectory represented the original one well concerning spatial shape and geographic information.

To reduce the interference of the time factor on the clustering process, all trajectories were equally divided to pick out the same number of points as samples. The distance matrix was then calculated between the corresponding sample points of each two trajectories. After that, a hierarchical agglomerative clustering algorithm was conducted, and the similarities between clusters were measured based on the complete linkage (Defays, 1977), i.e. calculating the distance between two most distant points in two clusters. Since this clustering process was unsupervised without knowing the optimal number of clusters, we evaluated the clustering quality using two common criteria, the average of the Sum of Squared Error (SSE) (Han et al., 2011) and the Silhouette Score (Rousseeuw, 1987) while varying the number of clusters, and determined the optimal results consequently.

**Fig. 5:**
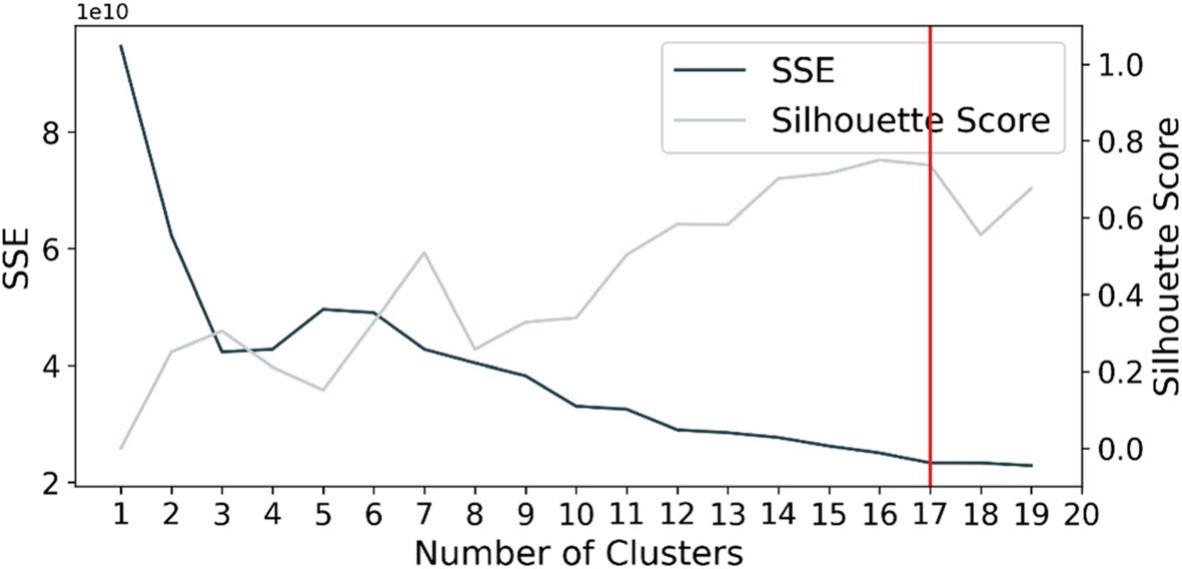
Clustering results' validation with SSE and Silhouette Score

**Fig. 6:**
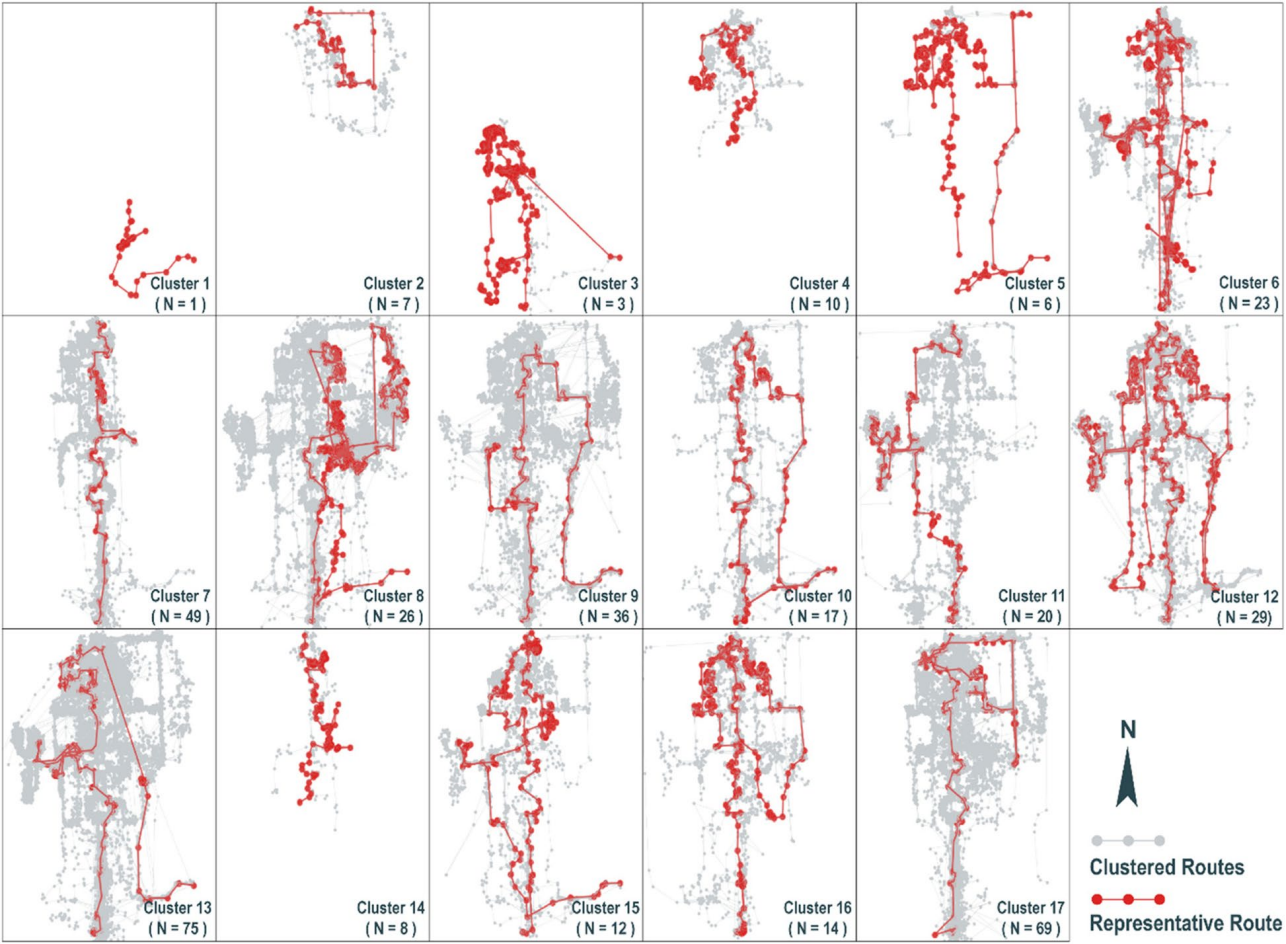
Clustering results and representative routes of raw-data-based approach

Figure 5 shows that the optimal number of clusters was 17, since a clustering result of good quality requires a smaller SSE and a larger Silhouette Score (greater than 0.3 is better). The clustering results with representative routes were visualized in Fig. 6. Seventeen clusters of trajectories showed distinctions regarding route complexity, the number, and the order of the attractions visited. The trajectories in clusters 1, 2, 3, 4, and 14 were not quite complete, probably due to the data deficiency caused by equipment malfunction, indicating that the outliers could be well recognized using this clustering method. Clusters 7, 13, and 17 represented three major route types respectively, and the representative trajectories of the three clusters showed that the order of route complexity is as follows: cluster 7 < cluster 17 < cluster 13. It is noted that the plotted representative trajectories were not always that representative when the trajectories in the clustering category were rather dispersed, for example from a geographic perspective, the most evident feature in clusters 8 and 9 was the northeastern part of the trajectories, but it did not appear in the representative route in cluster 9, and the westernmost area which featured in cluster 8 was not shown in the representative route, either. This is because the representative routes were calculated and selected via the program automatically, and characterized by a minimum sum of distances to the other trajectories within their clusters. It was therefore difficult to represent all features of a cluster with a single trajectory, particularly when the trajectories were complex.

The clustering results indicate that although the spatial similarities of trajectories were discovered using this approach, the information provided by such clustering results was vague for complex trajectories. Besides, although there were available methods to help determine the optimal number of clusters, the changes in sample size could strongly affect the stability of the clustering results, making it difficult to draw conclusions that could be generalized to other places. Another weakness of this approach was that the running time of the algorithm was rather lengthy. Nevertheless, the raw-data-based trajectory mining approach was shown to be effective for experimental exploration of newly collected unknown datasets, quick understanding of trajectories’ components, and identification of the outliers that cannot be easily recognized in pre-processing.

### Sequence-based approach: common routes identification from a semantic perspective

**Fig. 7:**
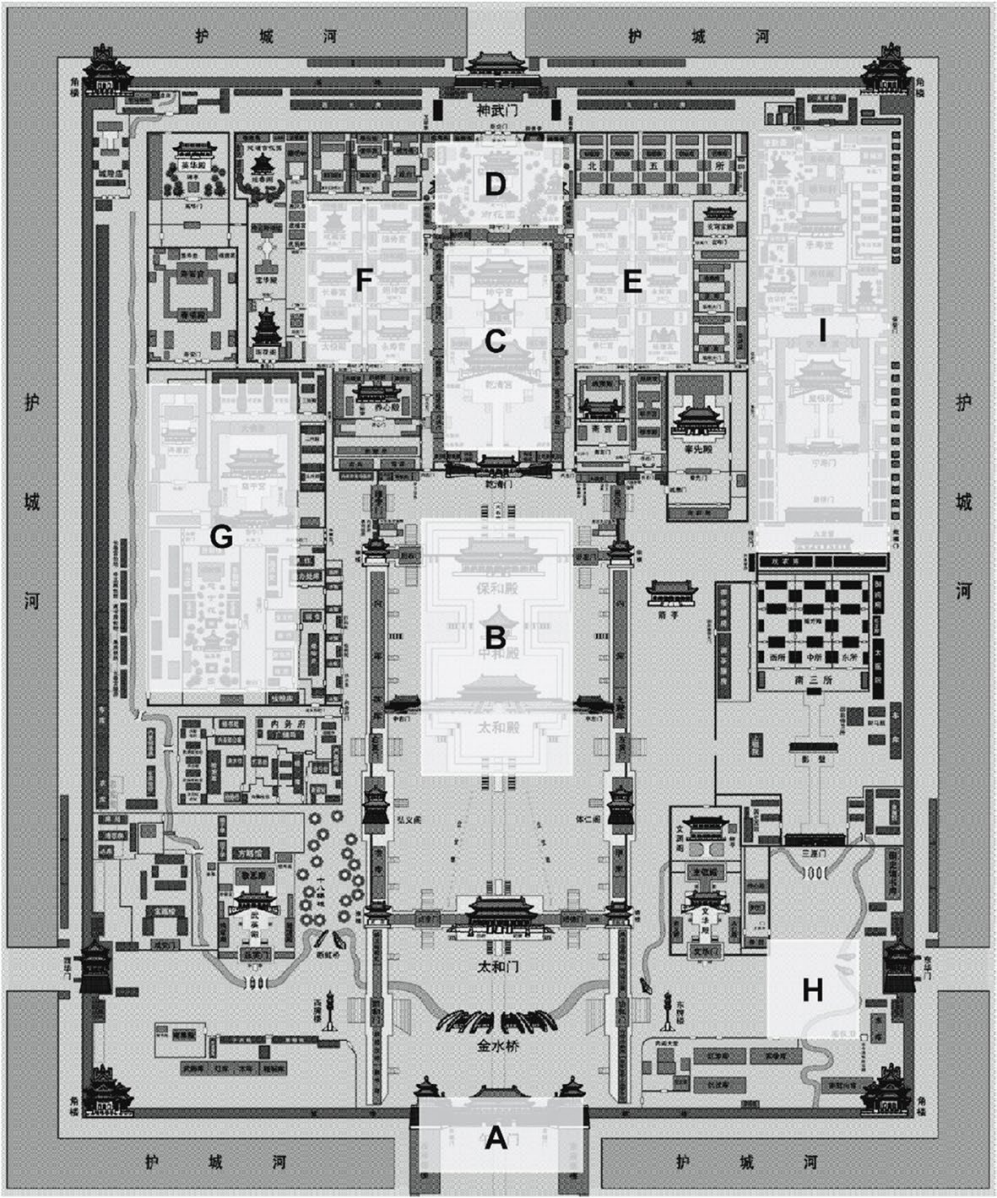
Coding eight main attraction areas and a secondary exit of the Palace Museum

To differentiate from the previous experiment, this experiment used a supervised method for the test of sequence-based approaches, aiming at identifying the common routes chosen by the visitors from a semantic perspective. Following the pre-processing procedure, we converted all cleaned trajectories and ten recommended tour routes used as alignment references into sequences of attraction areas for pairwise alignment. Eight main attraction areas with a secondary exit were defined and coded according to the original zoning of the Palace Museum, as shown in Fig. 7. The recommended tour routes were classified into three categories (short, medium, and long routes) according to estimated time length, and relative information including coded sequences, route description, and route code is presented in Table 1.

**Table 1:** Ten recommended tour routes for the Palace Museum

No.	Sequence	Route Description	Route Code
1	ABCD	Fast Tour 1: Central axis	S1
2	ABFD	Fast Tour 2: Palaces with original status exhibition	S2
3	ABGH	Fast Tour 3: Southern part only	S3
4	ABCFD	Half-Day Tour 1: Central axis with western six palaces	M1
5	ABCEI	Half-Day Tour 2: Eastern part only	M2
6	ABCDH	Half-Day Tour 3: Central axis with southeast exit	M3
7	ABGCFD	Half-Day Tour 4: Western part only	M4
8	AGDEI	Half-Day Tour 5: Flower viewing	M5
9	ABCFDEI	One-Day Tour 1: All attractions without the Cining Garden	L1
10	AGFCDEBI	One-Day Tour 2: All attractions	L2

**Fig. 8:**
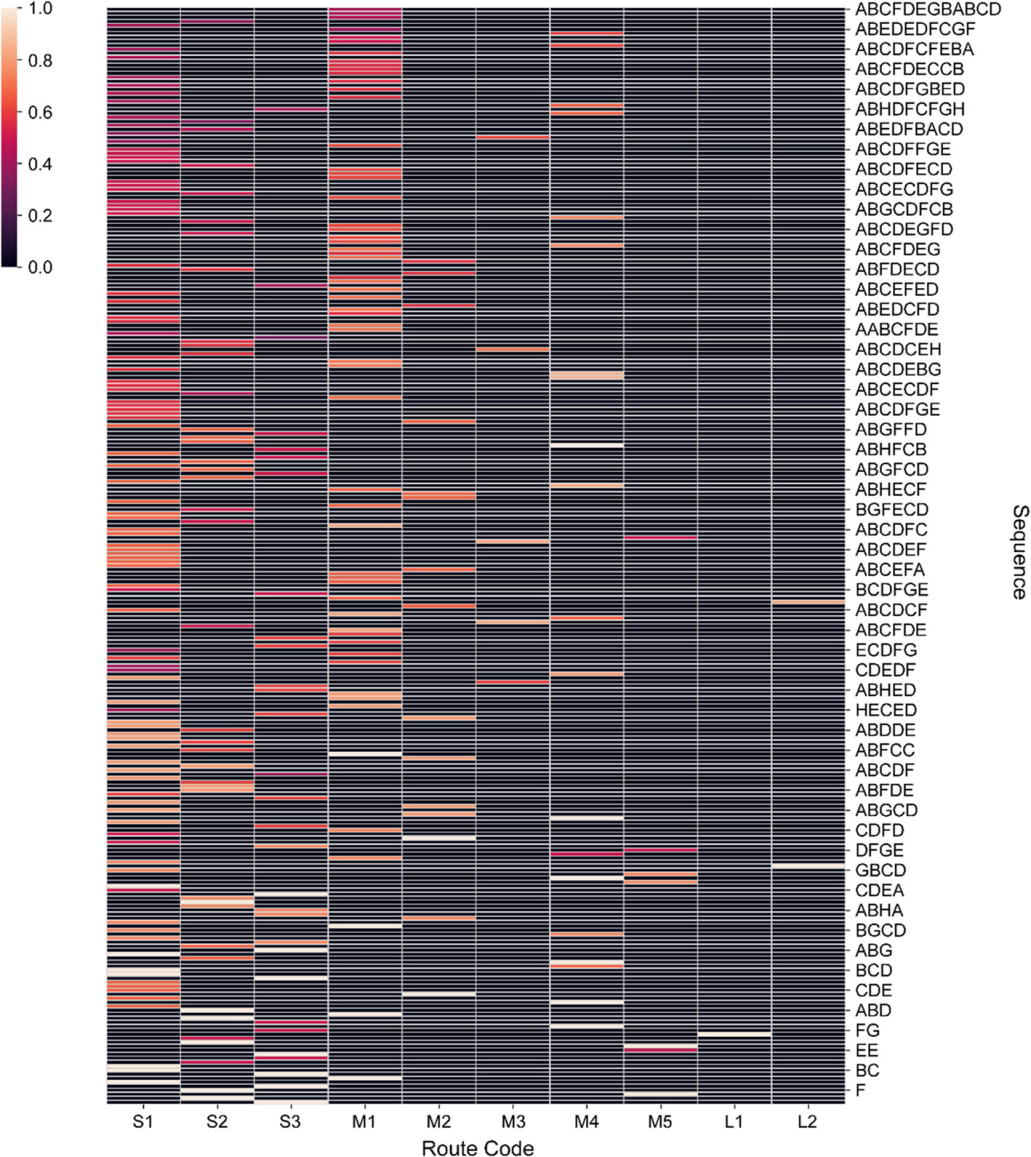
Distribution of matching scores between trajectory sequences and recommended routes

The sequence alignment method in this study was implemented with a Python package called Bio, which contains a specialized function to compute match scores between sequences, and the match score matrix was computed between each trajectory sequence and each pre-defined route sequence accordingly. The thermal distribution of all sequence alignment results is shown in Fig. 8. The brighter the color, the higher the matching score is. It should be noted that a 100% match does not mean that the two sequences are exactly the same, for example when aligning a short sequence with a long sequence, if the short sequence is the same as part of the long sequence, the matching score will also be high.

As shown in Fig. 8, route S1 and route M1 had the largest numbers of matching results with high matching scores, which covered both short and long trajectory sequences, indicating that these were two mostly frequent subsequences in all trajectory sequences. Route S1 corresponded to the sequence “ABCD”, representing the complete central axis of the Palace Museum. With respect to a complex trajectory sequence, it is hard to match a route with a same sequence, but the trajectory likely contains the most important route of the central axis. Regarding route M1, its corresponding sequence “ABCFD” had an additional western area “F” relative to the central axis, indicating that it was a common visiting route in the Palace Museum chosen by a majority of visitors relative to the eastern route. The numbers of matching results for routes L1, L2, and M5 were rather small, in that routes L1 and L2 were too long to match identical trajectory sequences, and route M5, as seen in Table 1, was a less common route specially designed for visitors with particular objectives. This finding suggests that visitors with sufficient time budget would arrange their tour routes more freely rather than strictly following any recommended routes.

The alignment results demonstrate the effectiveness of the sequence-based approach, which well identified frequent subsequences that represented the core routes, and uncovered both the flexibility and fidelity characteristics of the trajectories. To address research questions of diverse granularity, the sequence coding could be altered to extend the applicability of the method. If other information, including visitors’ tour duration and demographic characteristics, were analyzed for inclusion, the tendency of route selection under different initial conditions could be further explored and applied to tasks including trajectory prediction and route recommendation, which is significant for flow design and spatial functional planning.

### Metrics-based approach: movement behavioral pattern recognition from a quantitative perspective

This experiment selected three basic metrics, including total distance, total duration, and average speed, to describe the movement characteristics of trajectories, and used them as a feature matrix for trajectory clustering. Since all three metrics were approximately Gaussian distributed, a Gaussian Mixture Model (GMM) using the Expectation-Maximization (EM) framework (Xu & Jordan, 1996) was performed as the clustering algorithm. The feature matrix was considered a mixture of multiple Gaussian distributions, and the components of these Gaussian distributions best reproducing the original data are the possible clustering results. GMM is a probabilistic-density-based algorithm, and therefore, two criteria, the Akaike information criterion (AIC) and the Bayesian Information Criterion (BIC) were utilized to test the performance of the fit model. These two criteria helped determine the optimal number of components, and the smaller the AIC/BIC value, the better the model’s performance. In addition, this paper used the Silhouette score as a double validation for the quality of the clustering results.

**Fig. 9:**
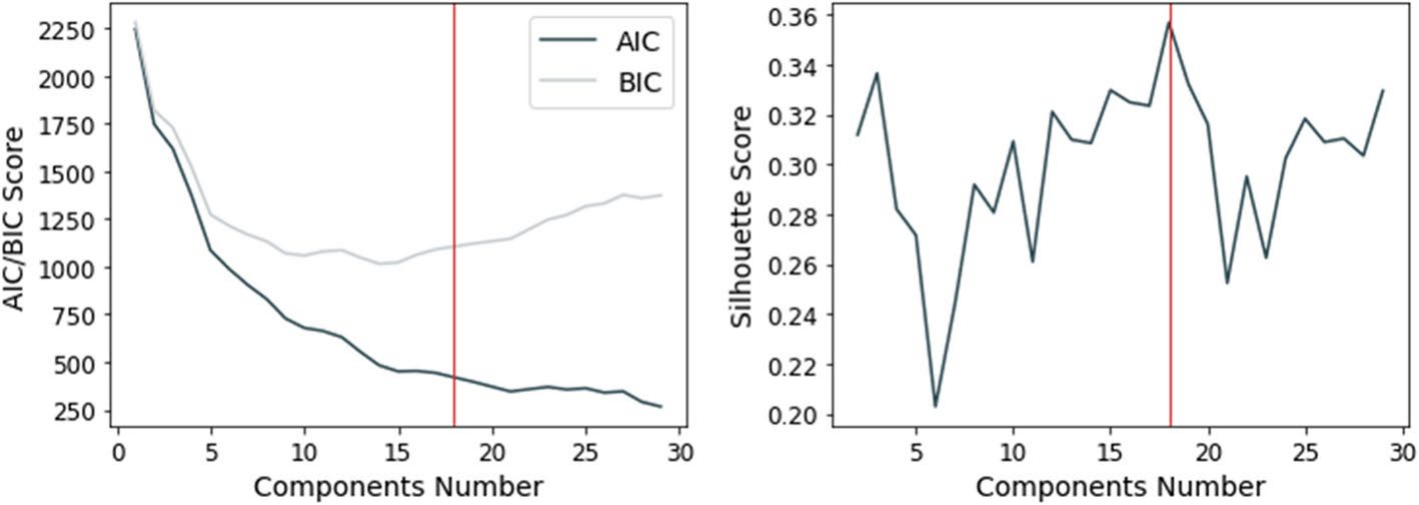
Clustering results’ validation with AIC, BIC, and Silhouette Score

**Fig. 10:**
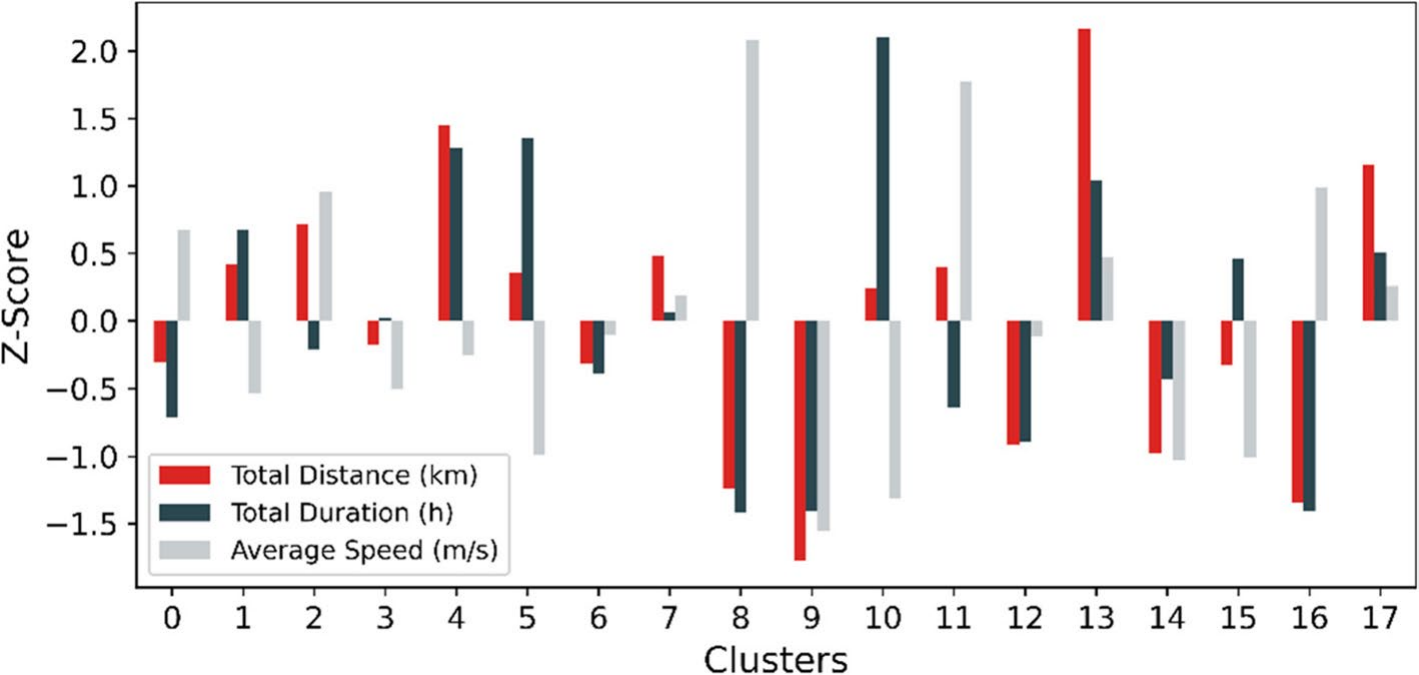
Movement characteristics of 18 clusters

The result of the validation process of clustering model quality by varying the number of components is shown in Fig. 9, and according to three criteria, the optimal number of clusters was 18. The features of 18 clusters were described using the three measures normalized using the z-score method, as shown in Fig. 10, and a positive or negative value indicates whether the result was above or below the average. Several interesting results were observed, for example, visitors in cluster 13 walked far longer distances relative to the average, but the total duration and average speed were distributed only slightly greater than the average level, not particularly outstanding; while the total duration of cluster 10 exceeded the average by far, but the distance traveled was close to the average length and the average speed was rather slow.

**Fig. 11:**
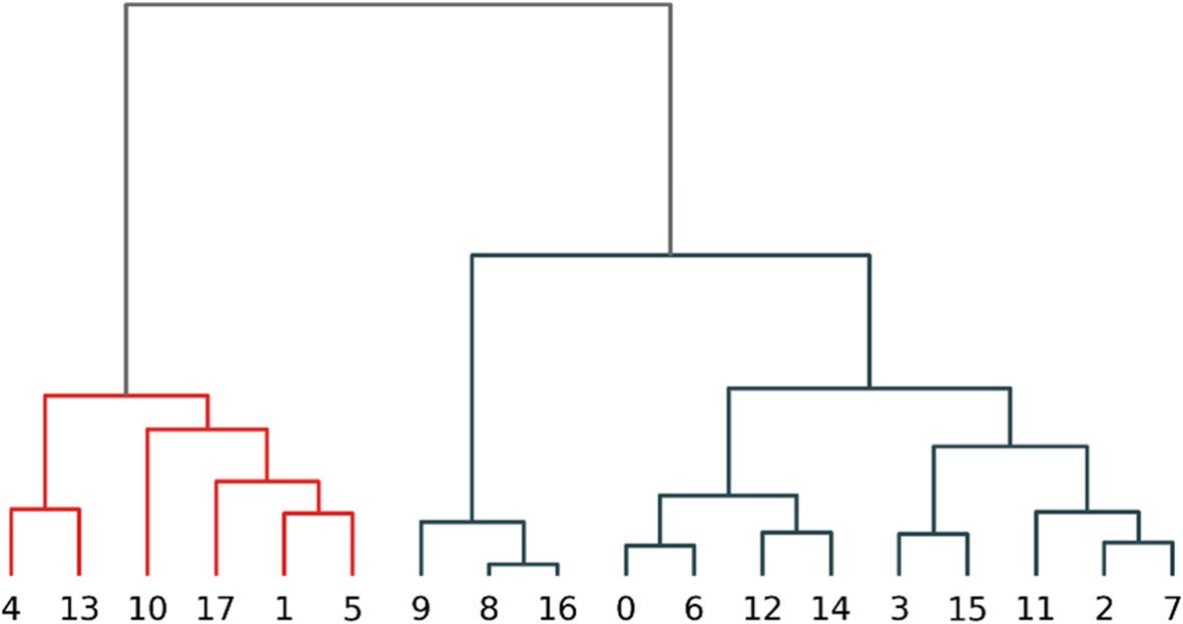
Dendrogram of clustering results

A dendrogram for the 18 clustering results to better observe their categorization and affiliation relationships was calculated and drawn in Fig. 11. The result shows that higher tiers in the hierarchy were divided based on the consistency and the closeness to the average of the distance and duration measures, and lower-tier division was based on the scale of the average speed. Clusters 9, 8, and 16, for example, shared common features that both distance and duration were much smaller relative to the average level and split over the feature of average speed on the next level, where the speed of clusters 8 and 16 was much faster relative to the average level and cluster 9 was much slower. The consistency of distance and time indicates whether the visitors kept walking or strolled with stopovers, which might be related to the physical strength of the visitors and the purpose of the tour. To gain relevant conclusions requires further analysis of the clustering results in association with other data regarding human characteristics and spatial features.

As the objective of this experiment was only to verify the effectiveness of the metrics-based approach, it did not dig deeper. Nevertheless, the method has shown great potential due to the good interpretability and high quantification of the clustering results. These uncovered movement behavioral patterns will not lose their meanings as the study site changes, and if supplemented with other semantic information, we could figure out why and how these patterns are formed and discover the associations between human behavioral characteristics, subjective psychology, and other objective influencing factors. These findings could also provide higher dimensional, more quantitative, and more universal strategies for flow design.

## Discussion and conclusion

This article presented a systematical review of three categories of trajectory mining approaches (i.e. raw-data-based, sequence-based, and metrics-based approaches) and examines the applicability and scope of the three dimensions of the mining approaches, respectively. The results of this study indicate that mass visiting behavior patterns do exist in the Palace Museum, and these patterns were reflected in frequent visiting areas, visiting orders, route planning or selection, and spatio-temporal resource allocation. In other words, it is feasible to employ complex human trajectory data to enhance the understanding of the way people use the built environment and space.

The study aimed to show the applicability and potential contribution of trajectory mining approaches for behavioral analysis. As a result, specific results concerning visitors to architectural sites with similarities to the Palace Museum were limited since extensive explorations of each experiment would be beyond the capability of a single paper. Nevertheless, it is still possible to gather behavioral insights into the great potential of such approaches and provide a wide range of implications. From a methodological point of view, the first and third experiments in this paper used unsupervised clustering methods, while the second experiment exploited an innovative supervised route matching method to classify trajectories by their similarities to the pre-designed routes. This method has not been used in previous studies in the fields of tourism or transportation, although its principle is rather simple. Yet, it is well suited for behavioral studies and flow design evaluation in architectural spaces, since many public buildings possess a sightseeing nature and hence pre-designed flow lines to guide the visitors and improve spatial efficiency. These empirical flow lines could be used as classification references when analyzing large and complex trajectory datasets. As a result, pre-designed routes with better matching performance could be considered an effective flow design, while trajectories with poor matching results could provide useful insights into individuals’ special demands for space usage.

Beyond the methodological insights, the results in this article have a more practical bearing on the reasonable allocation of public resources and could be extended to the intelligent control of building energy services. In this respect, mining trajectories from both a geographic and quantitative perspective are of great value. The geographic perspective could provide spatial and temporal distribution patterns of the users and help avoid operations that would cause waste of resources or energy, while clustering the users based on the movement metrics could facilitate the provision of efficient, energy-saving, and environment-friendly services for various user groups.

The mining approaches used in this study have the potential in uncovering the spatial-temporal behavioral patterns in the field of architecture, but which approach to adopt depends more on the problem to be solved. In general, the higher the dimensionality in utilizing the data, the more effective the approach is in discovering generalizable knowledge of human behavioral patterns. The raw-data-based approach is limited by its overly geographic nature, but it is well suited for addressing problems in a specific space and time, for example how to optimize the setting of environmental facilities in a park or a building complex exactly requires trajectory analysis of this granularity. If we, however, are interested in patterns that are not bound to a specific environment, including mining similar spatial structures, recognizing fixed activity patterns, etc., the sequence-based approach might provide more useful insights due to its semantic advantages. To raise the dimension of the problem, the important role of individuals using a space, including the social-demographic characteristics, psycho-emotional changes, goals, and motivations, cannot be ignored. A more generalized behavioral pattern that can be discussed on the same level as the above issues is then required to be discovered in complex trajectories, where a metrics-based approach from a fully quantitative perspective could be helpful and meaningful.

Another important issue with trajectory pattern mining concerns its relationship with conventional data types. The analyses in this paper revealed a segmentable feature of the visitors’ trajectories in the Palace Museum, yet questions such as how multiple trajectory types were generated and which individuals exhibited behavioral consistency are worth exploring in future studies. The current study showed that these questions regarding individual characteristics and relative semantic information like motivations cannot be achieved using GPS trajectory data alone. Xu et al. (2020) used a K-means clustering algorithm to summarize and classify visitors’ spatio-temporal behavior, whose basic idea was similar to the third experiment in this study; however, with a mixed-use of GPS trajectory data and a questionnaire, they further compressed seven clusters into two major spatial patterns and better explained the visitors’ behavior. East et al. (2017) also combined GPS and survey data to investigate the effects of the demographic factors exerted on visitors’ spatial and temporal behavior and successfully differentiated between the behaviors of different group types. GPS trajectory data and mining techniques could therefore not replace interviews, questionnaires, or field observations which contain important sources of information on human characteristics, psychological motivation, and spatial features (Shoval & Isaacson, 2007). Conversely, trajectory data should be mined along with semantic data to maximize its effectiveness and potential.

Despite the obvious value of GPS trajectory pattern mining, it does have several limitations. The accuracy of GPS, for example, decreases when used indoors, due to satellite signals being blocked by building structures such as reinforced concrete. From this point of view, trajectories collected from more accurate indoor positioning techniques like the mobile phone could be developed deeper in the field of behavioral studies related to the built environment. Other sources or tools for collecting big data positioning data including Wi-Fi IPS and UWB IPS could provide data approximate to trajectory data, and can be applied to the trajectory mining approaches mentioned in the article. In addition to positioning information, human feelings and emotional fluctuations while using the space have great value for research examining environmental psychology. The potential of new emotional monitoring technologies such as portable biometric information measurements should be verified and demonstrated in future research (Shoval et al., 2018).

Another area of importance yet to be explored is data-driven intelligent decision-making concerning architectural programming and smart design. The application of trajectory pattern mining in the context of architectural environments would allow researchers to establish a comprehensive database of human behavior related to the built environments along with spatial information. Typologies of spatial-temporal behavior could be useful in offering suggestions from the design phase and predicting the activity and movement behavior of people in a newly built spatial environment.

Overall, GPS trajectory data and trajectory mining approaches offer an effective means of discovering behavioral patterns from the spatial-temporal movement data, which, if appropriately applied, could push forward the boundaries of architecture studies. It is worth noting that trajectory mining could also contribute and provide insights to enhance public health and safety. In the current COVID-19 pandemic era, the changes in how people use public buildings and spaces are worthwhile to investigate, which might trigger an even wider enthusiasm for GPS trajectory studies.

## Data Availability

The datasets generated during and analyzed during the current study are not publicly available due to them containing information that could compromise research participant consent but are available from the corresponding author on reasonable request.
